# Assessing cetacean surveys throughout the Mediterranean Sea: a gap analysis in environmental space

**DOI:** 10.1038/s41598-018-19842-9

**Published:** 2018-02-15

**Authors:** Laura Mannocci, Jason J. Roberts, Patrick N. Halpin, Matthieu Authier, Oliver Boisseau, Mohamed Nejmeddine Bradai, Ana Cañadas, Carla Chicote, Léa David, Nathalie Di-Méglio, Caterina M. Fortuna, Alexandros Frantzis, Manel Gazo, Tilen Genov, Philip S. Hammond, Draško Holcer, Kristin Kaschner, Dani Kerem, Giancarlo Lauriano, Tim Lewis, Giuseppe Notarbartolo di Sciara, Simone Panigada, Juan Antonio Raga, Aviad Scheinin, Vincent Ridoux, Adriana Vella, Joseph Vella

**Affiliations:** 1 Marine Geospatial Ecology Laboratory, Nicholas School of the Environment, Duke University, Durham, NC 27708 USA; 2 UMR MARBEC (IRD, Ifremer, Université de Montpellier, CNRS), Institut Français de Recherche pour l’Exploitation de la Mer, Avenue Jean Monnet, CS 30171, 34203 Sète, France; 3 Observatoire PELAGIS UMS 3462 Université de La Rochelle/CNRS, 5 allées de l’Océan, 17 000 La Rochelle, France; 4 Marine Conservation Research (MCR), 94 High Street, Kelvedon, CO5 9AA UK; 5 Song of the Whale research team, International Fund for Animal Welfare (IFAW), 87-90 Albert Embankment, London, SE1 7UD UK; 6 Institut National des Sciences et Technologies de la Mer (INSTM), Centre de Sfax, B.P. 1035, Sfax, 3018 Tunisia; 7 Alnilam Research and Conservation, Pradillos 29, 28491 Navacerrada, Madrid Spain; 8 SUBMON - Marine Environmental Services, Rabassa, 49, 08024 Barcelona, Spain; 9 EcoOcéan Institut, 18 rue des Hospices, 34090 Montpellier, France; 10 Italian National Institute for Environmental Protection and Research (ISPRA), via Vitaliano Brancati 60, 00144 Rome, Italy; 11 Pelagos Cetacean Research Institute, Terpsichoris 21, 16671 Vouliagmeni, Greece; 12 Morigenos - Slovenian Marine Mammal Society, Kidričevo nabrežje 4, 6330 Piran, Slovenia; 13 Department of Biodiversity, Faculty of Mathematics, Natural Sciences and Information Technologies, University of Primorska, Glagoljaška 8, 6000 Koper, Slovenia; 14 Sea Mammal Research Unit, Scottish Oceans Institute, University of St Andrews, St Andrews, Fife, KY16 8LB Scotland UK; 15 Blue World Institute of Marine Research and Conservation, Kaštel 24, HR-51551 Veli Lošinj, Croatia; 16 Croatian Natural History Museum, Demetrova 1, 10000 Zagreb, Croatia; 17 Department of Biometry and Environmental Systems Analysis, Albert-Ludwigs University Freiburg, Tennenbacher Straße 4, 79106 Freiburg i. Br., Germany; 18 Israel Marine Mammal Research & Assistance Center, Institute of Maritime Studies, School of Marine Sciences, The University of Haifa, Mt Carmel, 31095 Haifa Israel; 19 North Atlantic & Mediterranean Sperm Whale Catalogue (NAMSC), London, United Kingdom; 20 Tethys Research Institute, Acquario Civico, Viale G.B. Gadio 2, 20121 Milano, Italy; 21 Unidad de Zoología Marina, Instituto Cavanilles de Biodiversidad y Biología Evolutiva, University of Valencia, Aptdo 22085, 46071 Valencia, Spain; 22 The Morris Kahn Marine Research Centre, The University of Haifa, Haifa, Israel; 23 Centre d’Etudes Biologiques de Chizé (CEBC), UMR 7372 Université de La Rochelle/CNRS, 2 avenue Olympe de Gouges, 17000 La Rochelle, France; 24 Conservation Biology Research Group, Department of Biology, University of Malta, Msida, MSD2080 Malta; 25 The Biological Conservation Research Foundation, BICREF, PO BOX 30 Hamrun Malta; 26 Department of Computer Information Systems, University of Malta, Msida, MSD2080 Malta

## Abstract

Heterogeneous data collection in the marine environment has led to large gaps in our knowledge of marine species distributions. To fill these gaps, models calibrated on existing data may be used to predict species distributions in unsampled areas, given that available data are sufficiently representative. Our objective was to evaluate the feasibility of mapping cetacean densities across the entire Mediterranean Sea using models calibrated on available survey data and various environmental covariates. We aggregated 302,481 km of line transect survey effort conducted in the Mediterranean Sea within the past 20 years by many organisations. Survey coverage was highly heterogeneous geographically and seasonally: large data gaps were present in the eastern and southern Mediterranean and in non-summer months. We mapped the extent of interpolation *versus* extrapolation and the proportion of data nearby in environmental space when models calibrated on existing survey data were used for prediction across the entire Mediterranean Sea. Using model predictions to map cetacean densities in the eastern and southern Mediterranean, characterised by warmer, less productive waters, and more intense eddy activity, would lead to potentially unreliable extrapolations. We stress the need for systematic surveys of cetaceans in these environmentally unique Mediterranean waters, particularly in non-summer months.

## Introduction

A gap analysis—defined here as the process of assembling various datasets over a desired study area to identify where or when knowledge is lacking—is the first important step towards the development of large-scale species distribution models (SDMs)^1–5^. In the marine realm, data collection is particularly difficult, resource intensive and expensive^6^. As a result, our knowledge of mobile marine species distributions and densities is far from complete, underlining the need for gap analyses across large ocean basins. Gap analyses of mobile marine species observation data have led to identification of geographic areas and seasons with important knowledge gaps^7,8^. For example, a global gap analysis of line transect surveys used to derive abundance estimates and habitat-based density models of cetaceans has revealed large geographic gaps in the Southern Hemisphere and seasonal gaps in non-summer months^8^.

Assessing the geographic and seasonal coverage of species observation datasets is informative but not sufficient to assess the feasibility of SDMs, which typically rely on inferred species-environment relationships to derive predictions. It is critical to assess the coverage of datasets in environmental space to evaluate the extent of extrapolation when a SDM calibrated on existing data is used for prediction across a study region. Extrapolation in environmental space can lead to highly uncertain predictions because species-environment relationships are unknown in unsampled environments^9,10^. The extent of extrapolation has traditionally been visualized with environmental envelopes based on the ranges of individual covariates spanned by the data^10–12^. However, approaches based on such univariate envelopes fail to detect combinations of covariates that are within the univariate environmental space but outside the multivariate environmental space^13^. An analysis of datasets’ coverage in multivariate environmental space can flag potentially unreliable extrapolations resulting from these novel combinations^10,14,15^.

**Figure 1:**
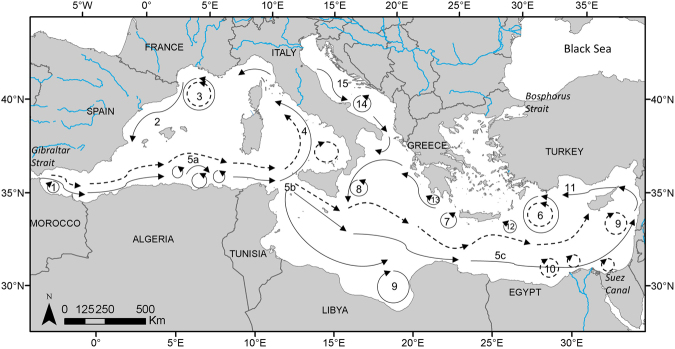
Map of main surface currents and gyres in the Mediterranean Sea. Dashed arrows represent summer circulation, plain arrows represent winter circulation. (1) Western Alborán gyre; (2) Ligurian-Provençal current; (3) Lions Gyre; (4) Thyrrhenian cyclonic circulation with summer weakening and eastern anticyclone; (5a) Algerian current and eddies, (5b) Atlantic Ionian stream and (5c) mid-Mediterranean jet; (6) Rhodes gyres; (7) Western Cretan gyre; (8) Western Ionian gyre; (9) Gulf of Sirte anticyclone; (10) Shikmona and Mers a-Matruh gyres; (11) Cicilian and Asia Minor current; (12) Iera-Petra gyre; (13) Pelops gyre; (14) Southern Adriatic gyre; (15) Western Adriatic coastal current. Figure adapted from Pinardi and Masseti (2000^52^. The map was generated with ArcGIS (http://desktop.arcgis.com/en/) (version 10.2.2).

The Mediterranean Sea (Fig. 1) is unique among large sea basins because it constitutes a miniature ocean with contrasted physical, climatic and biological characteristics^16^, and supports a highly diverse marine fauna including large mobile animals such as cetaceans^17,18^. The Mediterranean Sea biodiversity is undergoing profound alterations as high levels of anthropogenic pressures synergistically interact with the effects of climate change^19,20^. The manmade Suez Canal, which exposes the Mediterranean Sea to the distinct fauna of the Red Sea, caused the former to become the globally most invaded marine ecosystem^21^, augmenting and hastening biodiversity shifts. Despite these marked anthropogenic pressures, the collection of systematic data to assess marine animal abundances and responses to these stressors has been heterogeneous throughout the Mediterranean Sea, reflecting the uneven distribution of funding for population monitoring^22^. Cetacean population monitoring represents no exception: line transect survey programs to estimate cetacean abundances have been implemented mostly by European countries in the northwestern and central Mediterranean^23–26^. The wide range of some cetaceans across the Mediterranean Sea (e.g., fin whale and sperm whale^27–29^), combined with their vulnerability to the multiple anthropogenic pressures^30–33^, stress the need to develop predictive models to map their densities throughout the Mediterranean Sea.

As part of a regional-scale collaboration, we assembled for the first time line transect survey data collected across the Mediterranean Sea to identify gaps in the geographic, temporal, and environmental coverage of survey effort. Our objective was to evaluate the feasibility of mapping cetacean densities in the entire Mediterranean Sea by using models calibrated on available survey data and various environmental covariates. Our approach gives novel insights on traditional gap analyses solely based on spatiotemporal coverage, helps prioritise future survey efforts in the Mediterranean Sea, and is widely applicable to other marine regions and taxa.

## Results

### Spatiotemporal coverage of surveys

**Figure 2:**
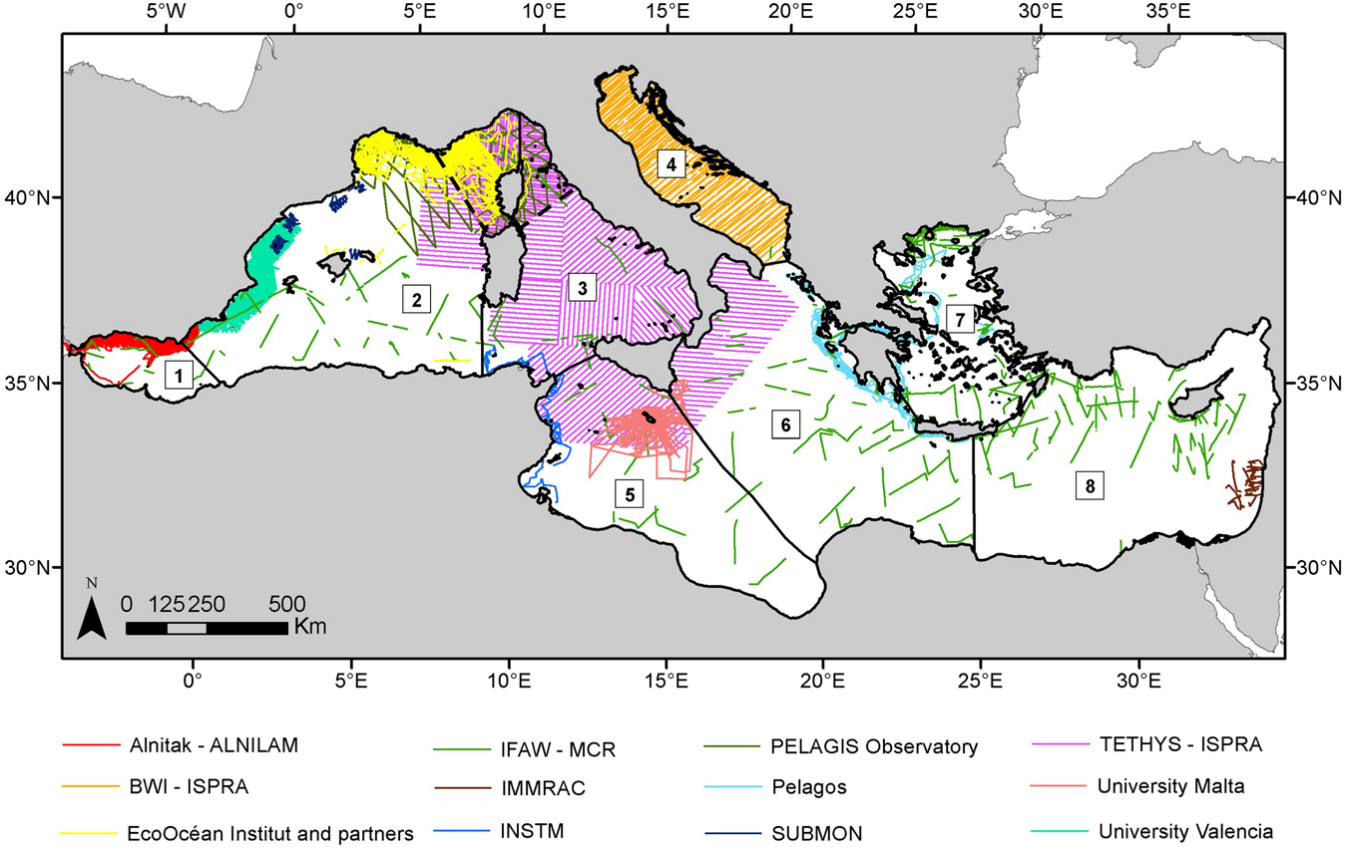
Line transect surveys in the Mediterranean Sea. Colours represent entities responsible for these surveys. Mediterranean subregions following Notarbartolo di Sciara (2016) and UNEP-MAP-RAC/SPA (2010)^18,56^: (1) Alborán Sea/Strait of Gibraltar, (2) Algero-Provençal Basin, (3) Tyrrhenian Sea/eastern Ligurian Sea, (4) Adriatic Sea, (5) Strait of Sicily/Tunisian Plateau/Gulf of Sirte, (6) Ionian Sea/Central Mediterranean, (7) Aegean Sea, (8) Levantine Sea. The location of the Pelagos Sanctuary^34^ is indicated with black dashed lines. Surveying entities: BWI = Blue World Institute of Marine Research and Conservation; ISPRA = Italian National Institute for Environmental Protection and Research; IMMRAC = Israel Marine Mammal Research and Assistance Center; INSTM = Institut National des Sciences et Technologies de la Mer; IFAW = International Fund for Animal Welfare; MCR = Marine Conservation Research. The map was generated with ArcGIS (http://desktop.arcgis.com/en/) (version 10.2.2).

We aggregated line transect surveys conducted by 12 organisations, including universities, consultancy companies and non-governmental organisations, resulting in a total of 302,481 km of effort (Table 1, Fig. 2). Aerial surveys represented 149,225 km of effort and shipboard surveys represented 153,256 km of effort.

**Table 1:** Details of Mediterranean line transect surveys incorporated in this gap analysis.

Surveying entities	Platform	Surveyed years	Surveyed subregion^1^	Total effort (km)	References
Alnitak – ALNILAM	Ship	1997–2011	Alborán Sea/Strait of Gibraltar	43,283	^33,66,67^
BWI – ISPRA	Aircraft	2010, 2013	Adriatic Sea	16,796	^68^
EcoOcéan Institut and partners^2^	Ship	1997–2002; 2005–2015	Algero-Provençal basin	52,608	^31^
IFAW – MCR	Ship	2003, 2004, 2005, 2007, 2013	Basin-wide	17,824	^45,69,70^
IMMRAC	Ship	2005	Levantine Sea	1,458	^46^
INSTM	Ship	2001, 2003, 2005	Strait of Sicily/Tunisian Plateau/Gulf of Sirte and Tyrrhenian Sea/eastern Ligurian Sea	2,352	^71^
PELAGIS Observatory	Aircraft	2011, 2012	Algero-Provençal basin and Tyrrhenian Sea/eastern Ligurian Sea	32,592	^26^
Pelagos Cetacean Research Institute	Ship	2001–2014	Ionian Sea/Central Mediterranean and Aegean Sea	16,742	^72^
SUBMON Marine Environmental Services	Ship	2010 2011 2015	Algero-Provençal basin	2,951	^73–75^
TETHYS – ISPRA	Aircraft and ship	2008–2011; 2013, 2014, 2016	Algero-Provençal basin and Tyrrhenian Sea/eastern Ligurian Sea	64,795	^23,25,76–78^
CBRG, University of Malta^3^	Aircraft and ship	1997–2015	Strait of Sicily/Tunisian Plateau/Gulf of Sirte	24,704	^24,79,80^
University of Valencia	Aircraft	2000–2003; 2010, 2011, 2013	Algero-Provençal basin	26,376	^81,82^

Surveying entities: BWI = Blue World Institute of Marine Research and Conservation; CBRG = Conservation Biology Research Group; IFAW = International Fund for Animal Welfare; IMMRAC = Israel Marine Mammal Research and Assistance Center; INSTM = Institut National des Sciences et Technologies de la Mer; ISPRA = Italian National Institute for Environmental Protection and Research; MCR = Marine Conservation Research.

^1^Mediterranean subregions following previous studies^18,56^.

^2^Partners: École Pratique des Hautes Études, WWF-France, Swiss Cetacean Society, Cybelle Planète, Participe Futur and Fondation Nicolas Hulot.

^3^A selection of the aerial and shipboard survey data collected by CBRG around the Maltese Islands was used in this analysis. Thus, the reported 24,704 km of effort represents part of the actual aerial and shipboard survey effort.

**Figure 3:**
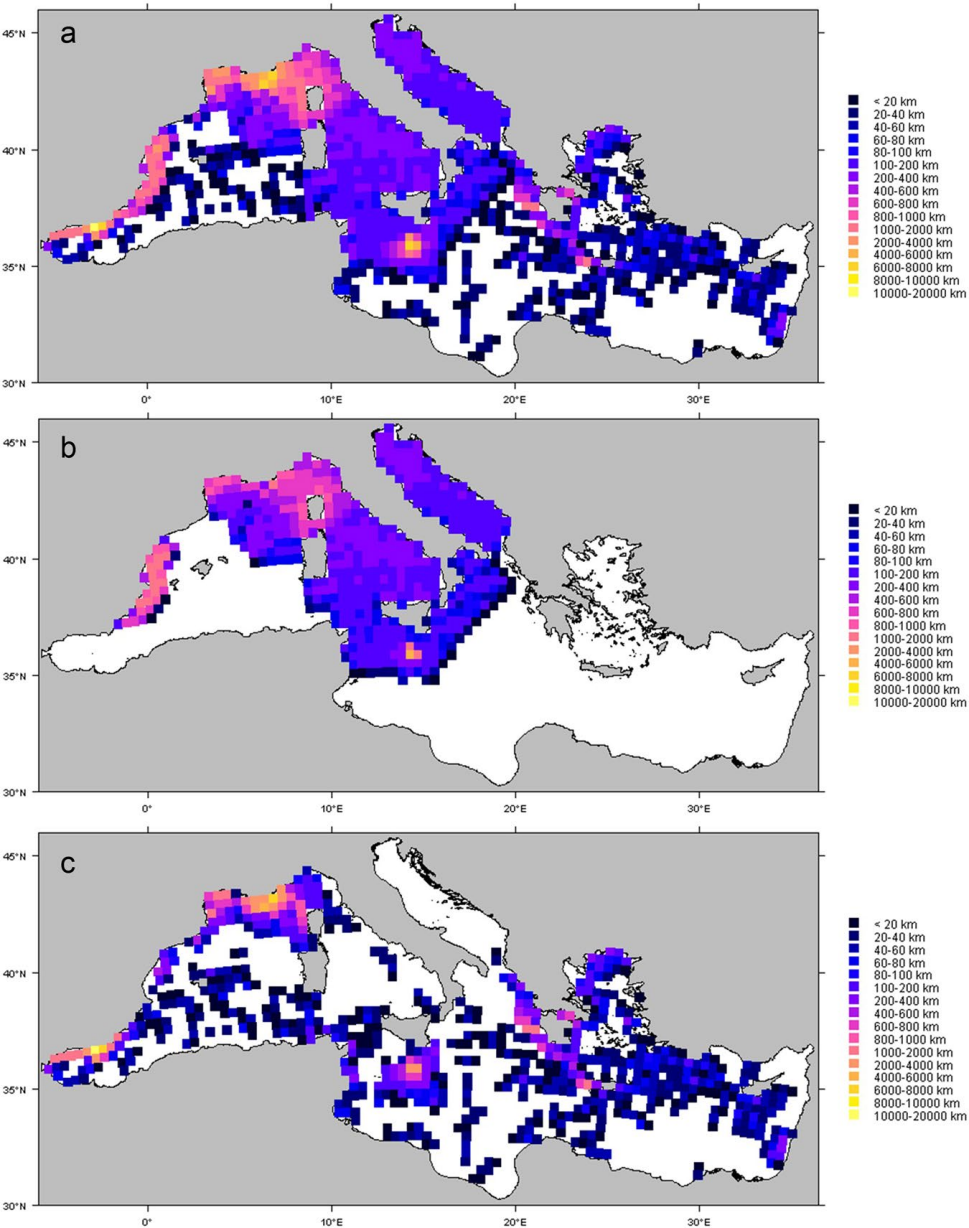
Geographic coverage of effort for: (**a**) all surveys, (**b**) aerial surveys only and (**c**) shipboard surveys only. Effort was aggregated on a 20 × 20 km grid for visualization (10 × 10 km cells used for the analysis were too small to be visible on a map of the entire Mediterranean Sea). The colour scale represents effort in km per 20 × 20 km grid cell and is the same for all three maps. Blank cells represent zero effort. The maps were generated with R (https://www.r-project.org/) (version 3.1.1).

Survey effort was concentrated in the northwestern Mediterranean and comparatively patchy in the eastern and southern Mediterranean (Fig. 3a). The Algero-Provençal basin was the Mediterranean subregion that received the largest amount of survey effort, followed by the Tyrrhenian Sea/eastern Ligurian Sea and the Alborán Sea/Strait of Gibraltar (Table 2). In comparison, the Levantine Sea and Aegean Sea subregions received only 2.1% of the effort each.

**Table 2:** Overall survey effort per Mediterranean subregion (defined following previous studies^18,56^).

Mediterranean subregion	Area (km^2^)	Area (%)	Effort (km)	Effort (%)
Alborán Sea/Strait of Gibraltar	62,134	2.5	38,415	13.2
Algero-Provençal basin	515,739	20.5	131,571	45.3
Tyrrhenian Sea/eastern Ligurian Sea	267,808	10.7	39,296	13.5
Adriatic Sea	133,364	5.3	16,204	5.6
Strait of Sicily/Tunisian Plateau/Gulf of Sirte	346,705	13.8	29,879	10.3
Ionian Sea/Central Mediterranean	497,523	19.8	22,482	7.7
Aegean Sea	187,984	7.5	6,214	2.1
Levantine Sea	501,476	20.0	6,127	2.1

Aerial surveys were conducted off Spain west of the Balearic Islands, in the Pelagos Sanctuary (a marine protected area for marine mammals in the Ligurian Sea established by international treaty^34^), in the Tyrrhenian Sea, in the Strait of Sicily (especially around the Maltese Islands), in the northern Ionian Sea, and in the Adriatic Sea (Fig. 3b). Shipboard surveys were conducted primarily in the northern Alborán Sea, in the western Ligurian Sea, in the eastern Ionian Sea, and around the Maltase islands. Shipboard survey effort was very patchy in the rest of the Mediterranean Sea (Fig. 3c).

**Figure 4:**
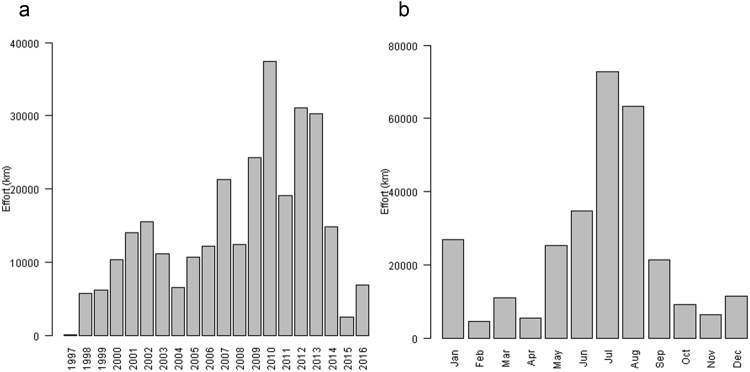
Overall survey effort (**a**) per year and (**b**) per month in the entire Mediterranean Sea for the study period (October 1997-April 2016). Note that years 1997 and 2016 did not include all months.

In terms of inter-annual survey coverage, two peaks were apparent: a small peak in the early 2000s and a larger peak between 2009 and 2013 (Fig. 4a) corresponding to the implementation of major aerial surveys. Survey effort was exceptionally low in 2015. The northern Alborán Sea, the northern Gulf of Lions and Ligurian Sea, the area around Maltese Islands, and the Hellenic Trench were covered by surveys in most years, reflecting long-term monitoring efforts (Supplementary Fig. S1).

In terms of intra-annual survey coverage, the amount of survey effort was largest in July and August and lowest in February and April (Fig. 4b). Most surveys were conducted from May to September. Survey effort was spread across the Mediterranean Sea from May to September, but restricted to the northwestern and central Mediterranean the rest of the year (Supplementary Fig. S2).

### Environmental coverage of surveys

#### Univariate environmental coverage

The surveys spanned broad ranges of values for all considered static covariates (depth, slope, distance to seamounts, and distance to canyons), resulting in limited univariate extrapolation throughout the Mediterranean Sea (Table 3) (Supplementary Figs S3 and S4).

**Table 3:** Spatial extent of extrapolation (i.e., the percentage of cells of the study area where extrapolation occurred) with single covariates and combinations of covariates. For dynamic covariates, the mean extent of extrapolation averaged over the 12 month period is provided, followed by the minimum and maximum monthly extents in parentheses. SST: sea surface temperature; PP: primary productivity; EKE: eddy kinetic energy.

Univariate extrapolation	
Depth	0.1%
Slope	0.0%
Distance to seamounts	0.0%
Distance to canyons	0.0%
SST	40.8% (0.5–40.8%)
PP	0.6% (0.0–21.0%)
EKE	0.1% (0–17.1%)
Multivariate extrapolation	
All static covariates (depth, slope, distance to seamounts, distance to canyons)	3.7%
All dynamic covariates (SST, PP, EKE)	49.9% (8.1–55.1%)
All static and dynamic covariates (depth, slope, distance to seamounts, distance to canyons, SST, PP, EKE)	80.1% (55.5–96.5%)

The surveys spanned narrower ranges of values for the considered dynamic covariates (sea surface temperature, primary productivity, and eddy kinetic energy), resulting in a larger univariate extrapolation (especially in non-summer months) (Table 3).

**Figure 5:**
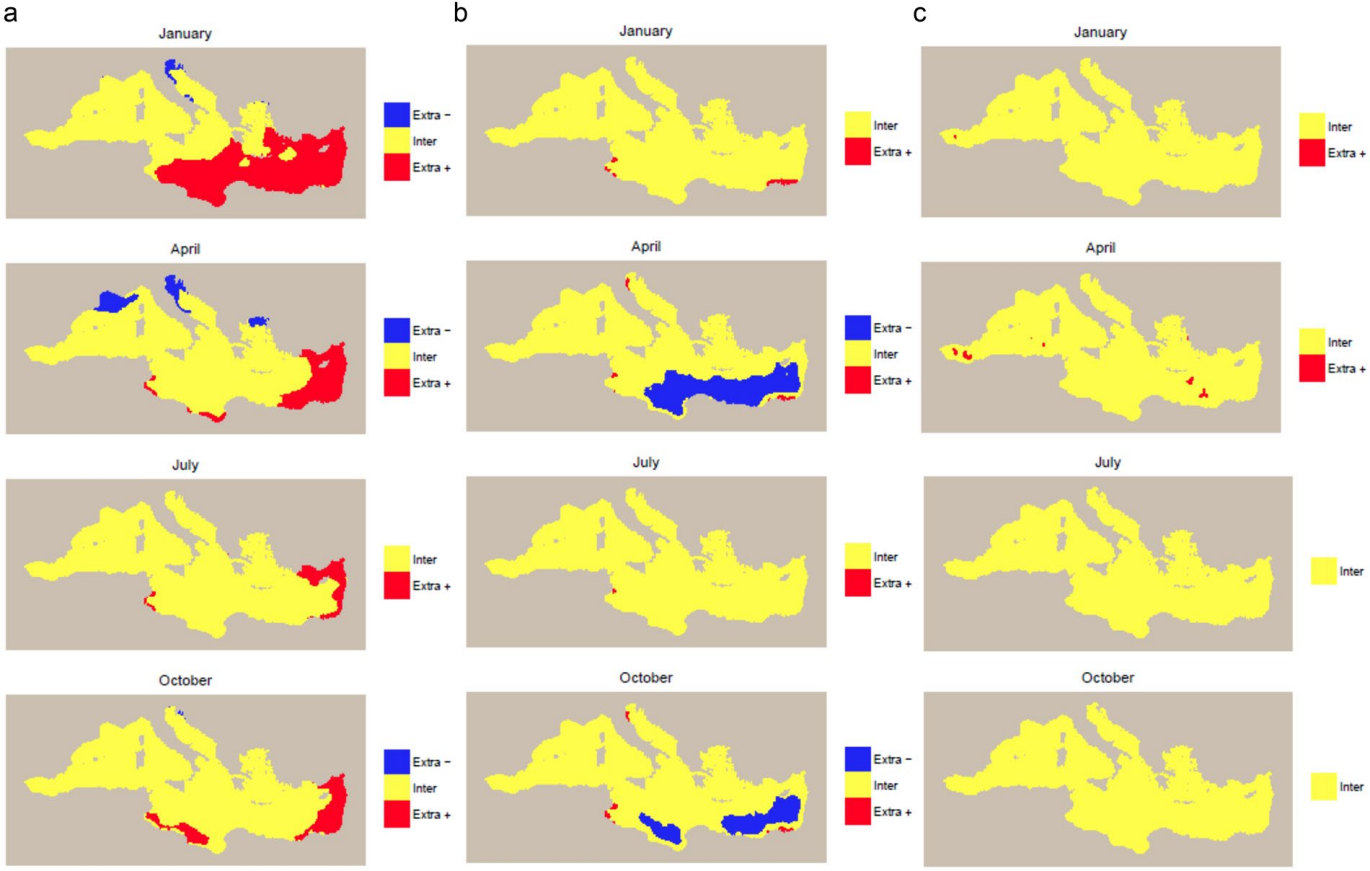
Extent of extrapolation *versus* interpolation if models calibrated on the available survey data were used for prediction across the Mediterranean Sea. (**a**) Model including sea surface temperature only; (**b**) model including primary productivity only; (**c**) model including eddy kinetic energy only. Cells where extrapolation to lower/higher values would occur are indicated in blue/red. Cells where interpolation would occur are indicated in yellow. Results for January, April, July, and October, corresponding to the middle month of solar seasons, are shown. Results for all months are shown in Supplementary Figs S6, S8 and S10. The maps were generated with R (https://www.r-project.org/) (version 3.1.1).

The extent of univariate extrapolation would be largest with the model including sea surface temperature only. In winter and spring, extrapolation would be needed to predict cetacean densities in warmer waters of the southeastern Mediterranean and in colder waters of the northern Mediterranean (Gulf of Lions, Adriatic Sea, and northern Aegean Sea) (Fig. 5a, Supplementary Figs S5 and S6). In summer and autumn, extrapolation would occur in warmer waters of the Levantine Sea and of the Tunisian Plateau (but with a reduced extrapolation compared to winter and spring).

Extrapolation in more productive coastal waters would occur year-round on the Tunisian Plateau and in non-summer months in the northern Adriatic Sea and off Egypt. In spring and autumn, extrapolation would occur in the less productive offshore waters of the southeastern Mediterranean (Fig. 5b, Supplementary Figs S7 and S8).

The extent of univariate extrapolation would be lowest with the model including eddy kinetic energy only. Extrapolation would occur in waters characterised by more intense eddy activity in the Alborán Sea and Levantine Sea, mostly in winter and spring (Fig. 5c, Supplementary Figs S9 and S10).

#### Multivariate environmental coverage

As expected, the extent of extrapolation was larger with combinations of covariates than with individual covariates (Table 3). For example, the extrapolation extent with the combination of sea surface temperature, primary productivity, and eddy kinetic energy was larger than the extrapolation extents with these three covariates individually.

**Figure 6:**
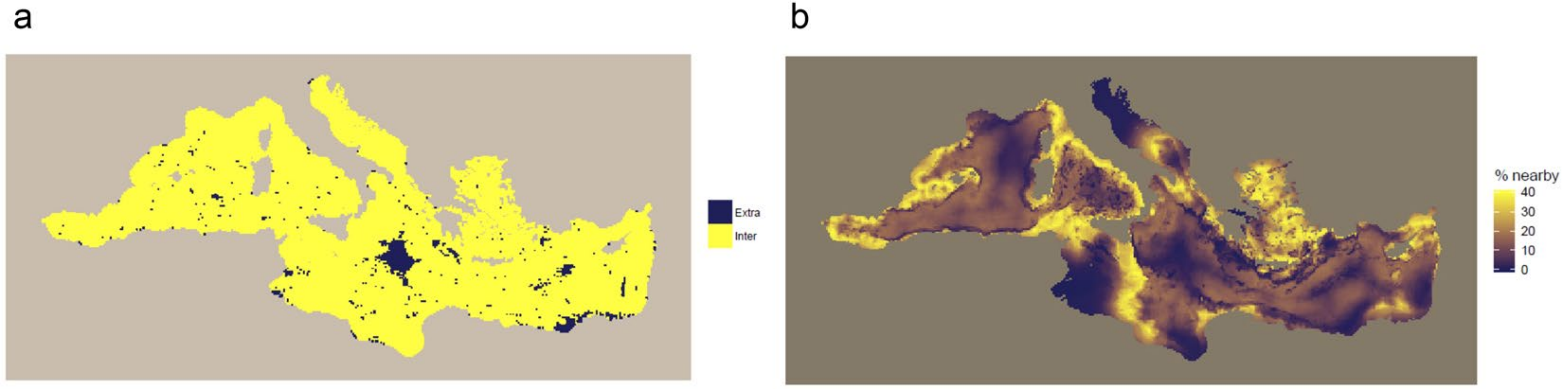
(**a**) Extent of extrapolation (dark blue) *versus* interpolation (yellow), and (**b**) proportion of prediction points near available data points in the multivariate environmental space defined by all considered static covariates if a model including all static covariates calibrated on the available survey data was used for prediction across the Mediterranean Sea. In (**b**), dark blue/yellow represents areas where predictions would potentially be unreliable/reliable. The definition of neighborhood in multivariate environmental space is provided in the Methods. The maps were generated with R (https://www.r-project.org/) (version 3.1.1).

For the model including all four static covariates, extrapolation would be limited to 3.7% of the Mediterranean Sea (Table 3). Extrapolation would mostly occur in offshore waters of the central Mediterranean and off Egypt (Fig. 6a). The proportions of data nearby in the multivariate space defined by static covariates were lowest on the Tunisian Plateau, in the northern Adriatic Sea, and in offshore waters throughout the Mediterranean Sea (Fig. 6b). Differences between extrapolation metrics (the extent of extrapolation and the proportion of data nearby) are presented in the Methods and discussed further below.

**Figure 7:**
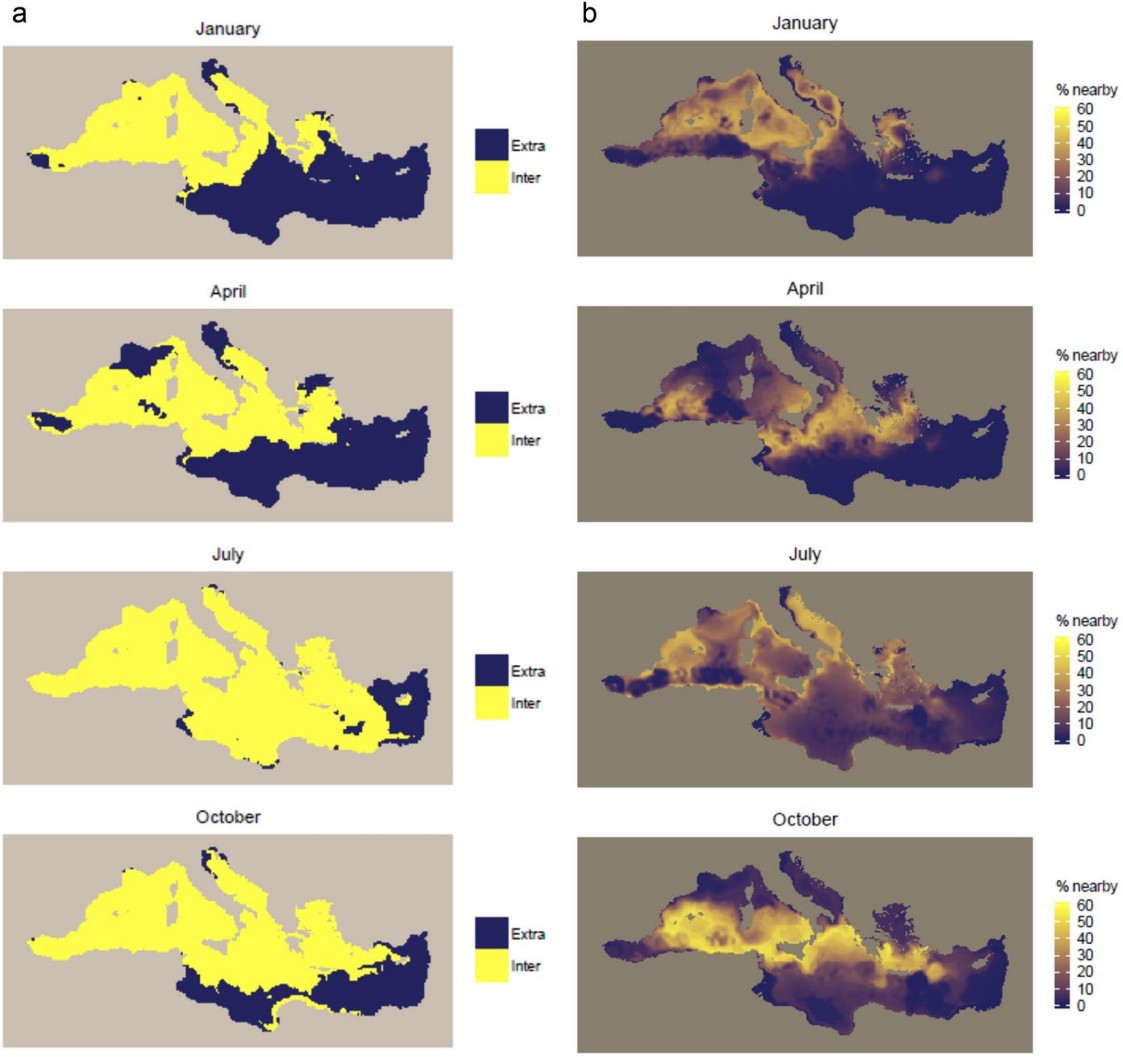
(**a**) Extent of extrapolation (dark blue) *versus* interpolation (yellow), and (**b**) proportion of prediction points near available data points in the multivariate environmental space defined by all considered dynamic covariates if a model including all dynamic covariates calibrated on the available survey data was used for prediction across the Mediterranean Sea. In (**b**), dark blue/yellow represents areas where predictions would potentially be unreliable/reliable. Results for January, April, July, and October, corresponding to the middle month of solar seasons, are shown. Results for all months are shown in Supplementary Figs S11 and S12. The definition of neighborhood in multivariate environmental space is provided in the Methods. The maps were generated with R (https://www.r-project.org/) (version 3.1.1).

For the model including all three dynamic covariates, extrapolation would occur in 49.9% of the Mediterranean Sea on average (minimum: 8.1% in September; maximum: 55.1% in February) (Table 3). Extrapolation would be widespread in the eastern Mediterranean and in parts of the western Mediterranean (e.g., the Alborán Sea and the Ligurian Sea), especially in winter and spring (Fig. 7a, Supplementary Fig. S11). In summer, extrapolation would be mostly limited to the Levantine Sea. The proportions of data nearby in the multivariate space defined by the three dynamic covariates were low in most of the eastern Mediterranean and in the northern and southern parts of the western Mediterranean (Fig. 7b, Supplementary Fig. S12). In autumn, higher proportions of data nearby appeared in a mid-latitude band. Some areas such as the Alborán Sea and the northern Adriatic Sea had low proportions of data nearby year-round.

**Figure 8:**
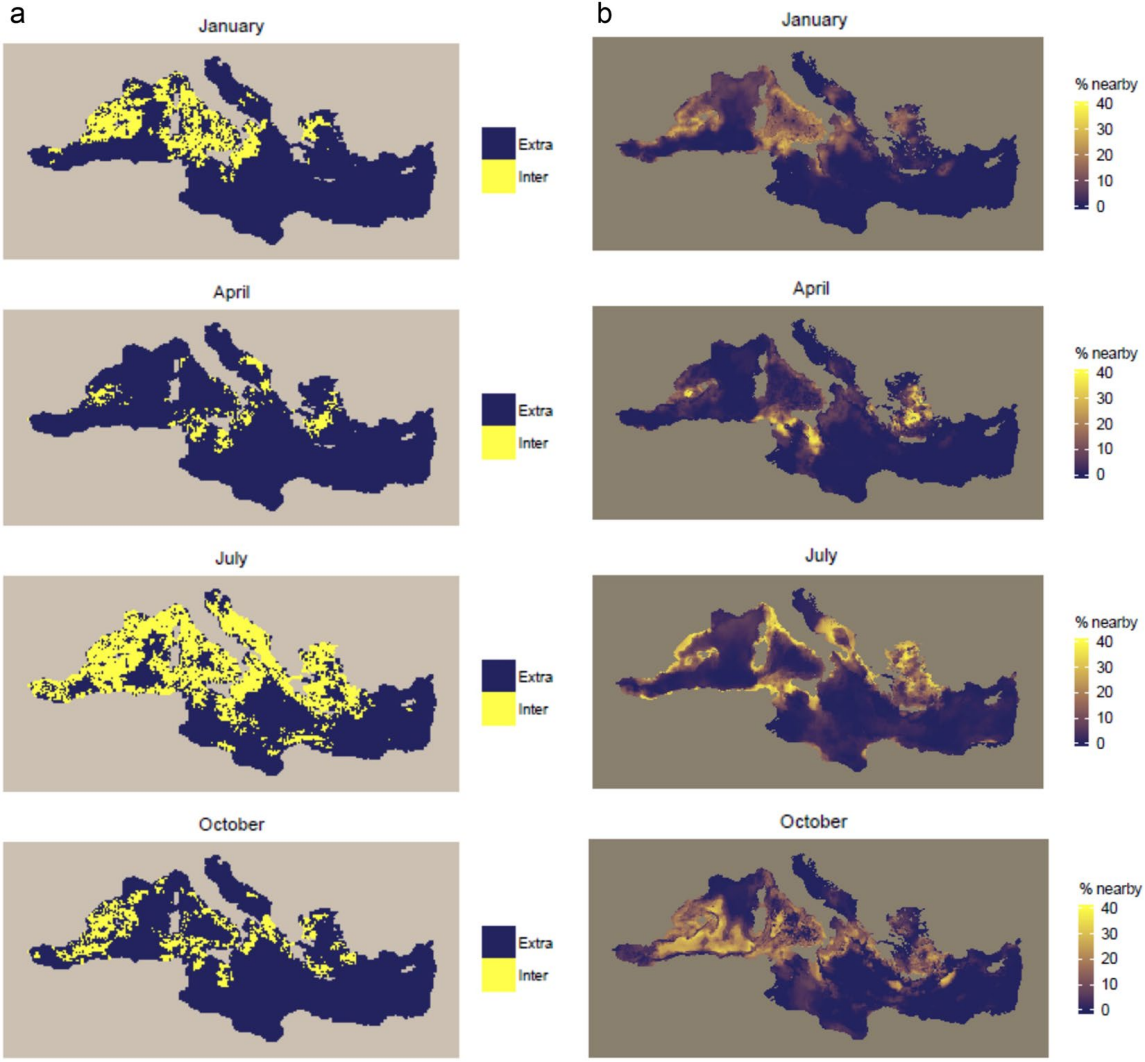
(**a**) Extent of extrapolation (dark blue) *versus* interpolation (yellow), and (**b**) proportion of prediction points near available data points in the multivariate environmental space defined by all considered static and dynamic covariates if a model including all static and dynamic covariates calibrated on the available survey data was used for prediction across the Mediterranean Sea. In (**b**), dark blue/yellow represents areas where predictions would potentially be unreliable/reliable. Results for January, April, July, and October, corresponding to the middle month of solar seasons, are shown. Results for all months are shown in Supplementary Figs S13 and S14. The definition of neighborhood in multivariate environmental space is provided in the Methods.The maps were generated with R (https://www.r-project.org/) (version 3.1.1).

For the model including all static and dynamic covariates, extrapolation would increase to 80.1% of the Mediterranean Sea on average (minimum: 55.5% in July; maximum: 96.5% in February) (Table 3). Extrapolation would be widespread in all seasons except in the western Mediterranean in summer (Fig. 8a, Supplementary Fig. S13). Overall, the proportions of data nearby in the multivariate space defined by all static and dynamic covariates were lower compared to the proportions of data nearby in the multivariate space defined by dynamic covariates only (as seen by the comparison of colour scales between Figs 8b and 7b). In winter and spring, the proportions of data nearby were low throughout the southeastern Mediterranean and in offshore parts of the western basin (Fig. 8b, Supplementary Fig. S14). In summer, the proportions of data nearby were overall higher in coastal areas, particularly to the north. In autumn, the proportions of data nearby were low in most of the eastern Mediterranean and in the northwestern Mediterranean.

## Discussion

We aggregated over 300,000 km of cetacean line transect surveys suitable for estimating abundance that were conducted in the Mediterranean Sea within the past 20 years by numerous organisations. Survey coverage was heterogeneous geographically and seasonally: large data gaps were present in the eastern and southern Mediterranean in all seasons and elsewhere in non-summer months. Survey coverage was also heterogeneous in environmental space. Surveys covered a number of static bathymetric features (e.g., coastal areas, continental slopes, submarine canyons and seamounts), but not the full range of dynamic oceanographic conditions found within the Mediterranean Sea. In particular, Mediterranean waters characterised by comparatively warmer temperatures, lower productivity and higher eddy activity were poorly surveyed for cetaceans. This raised the prospect that cetacean density models fitted to environmental covariates would have to be extrapolated in order to provide predictions for the entire Mediterranean Sea.

To assess extrapolation in environmental space, we mapped two metrics: the extent of extrapolation *versus* interpolation—a binary metric indicating whether a point is within the convex hull enclosing the sampled environmental space—and the proportion of data nearby in multivariate environmental space—a continuous metric derived from Gower’s distance. The first metric distinguishes predictions in unsampled geographic space that are within sampled environmental space (interpolation) from predictions in unsampled geographic *and* environmental space (extrapolation). It can be used to restrict predictions to the sampled environmental space by overlaying a mask on the prediction map or by flagging extrapolations in unsampled environmental space^13,35^; the standard errors of such extrapolations can then potentially be inflated until new data are acquired. The second metric provides a quantitative measure of the reliability of extrapolation in multivariate environmental space. It can be used as a measure of prediction uncertainty when maps of species distributions are needed for an entire region (e.g., to support management decisions). This metric is useful for differentiating predictions that are just outside the sampled environmental space from those that are far outside (and thus less reliable). It also identifies predictions that occur in sparsely sampled regions of environmental space despite being classified as interpolations by falling within sampled environmental space. For example, this situation was observed in the northern Adriatic Sea where low proportions of data nearby would occur although interpolation is predominant (see Fig. 6). Using the binary metric that distinguishes interpolation from extrapolation as the only measure of the reliability of predictions would lead to an overly high degree of confidence in predictions in the northern Adriatic Sea.

Mesgaran *et al*.^15^ proposed a modification of the Mahalanobis distance to assess deviations between the prediction and the calibration dataset using the mean and linear correlations between covariates^15^. We did not follow their approach because the covariates we selected *a priori* were not highly correlated (see Methods). Conn *et al*.^36^ assessed extrapolation using a model-based convex hull derived from a generalization of Cook’s independent variable hull. Because our study objective was to conduct a gap analysis of survey effort in environmental space (rather than to fit models), we adopted a model-independent approach.

We found that the extent of extrapolation was larger for combinations of covariates than for individual covariates. This is a common issue when predicting SDMs beyond the scope of the data: as more covariates are included in a model, new individual covariate values and new combinations of covariates appear, rapidly increasing the extent of extrapolation^13^. For example, in a study of small pelagic fish in the Gulf of Lions, models that included single covariates yielded less than 5% extrapolation while models that included five covariates exceeded 50% extrapolation^37^. For the Mediterranean cetacean surveys, a model that included all seven covariates resulted in 80% extrapolation; this model would be overly complex (in terms of having too many parameters) for reliably predicting cetacean densities at the regional scale. To predict species distributions or densities beyond the scope of the calibration data using SDMs, we recommend choosing models with a small number of covariates to limit the extent of extrapolation in environmental space.

Our analysis showed that the extent of extrapolation would be lower with static covariates than with dynamic covariates. Advantages of static covariates for modelling cetacean densities include generally low measurement errors and easy acquisition and processing leading to prompt model fitting. However, static covariates are rather indirectly related to cetacean densities. For example, steep seafloor slope may aggregate cetaceans via oceanographic processes that enhance prey availability and accessibility, assuming cetaceans are involved in foraging activities. Indirect ecological relationships are known to transfer poorly to new geographic regions because ecological processes underlying cetacean distributions may differ between surveyed and unsurveyed regions^38,39^.

By design, a gap analysis does not require the observation data (from which the response variable is derived) because it aims at assessing how representative sampling effort is of the environmental space where predictions are to be made. However, for the gap analysis to be useful, the environmental covariates to be tested should be the ones that are most likely to be selected when fitting the models. This can be done *a priori* (as in this study) by using sets of covariates that are ecologically-relevant and likely to be selected in future cetacean density models. This could also be done *a posteriori* for a given case study by using the set of covariates that has been actually selected in the model selection process. In this latter case, the response variable would be needed, not for the gap analysis itself, but to help identify the set of most relevant environmental covariates to be tested. When it comes to model development, it is important to keep in mind that (1) sample size of species observations affects model accuracy and precision^40^ and (2) the selected model strongly influences predictions, particularly when predicting outside the sampled range of the covariate data^41,42^.

Our study showed that if cetacean density models were calibrated on existing line transect surveys and predicted across the Mediterranean Sea, widespread extrapolations in environmental space would occur, leading to potentially unreliable predictions. However, it should be noted that the surveys available for this exercise were limited to those compatible with distance sampling, which is used to estimate absolute abundance. For certain management applications, relative abundance predictions will suffice. There are a number of additional surveys in the Mediterranean Sea that were not aimed at estimating absolute abundance with distance sampling but that followed similar methodology e.g.,^43,44^ and that could be included in models of relative abundance. Including such surveys would likely reduce some of the gaps in environmental space.

## Conclusion and Perspectives

Systematic survey efforts for cetaceans have previously been recognized as heterogeneous across the Mediterranean Sea^18,45,46^, but this heterogeneity was never quantified. By aggregating surveys conducted throughout the Mediterranean Sea, we were able to identify gaps in the spatiotemporal and environmental coverage of cetacean surveys for the first time.

We strongly recommend that future surveys be conducted in the eastern and southern Mediterranean where waters are characterised by warmer sea surface temperature, lower productivity, and higher eddy kinetic energy. These environmental conditions are found nowhere else in surveyed regions; they are unique to the eastern and southern Mediterranean and were under-represented in our dataset. Conducting surveys there would be particularly valuable in non-summer months because oceanographic conditions differ the most from those of other areas surveyed in those months. We also recommend additional survey effort in the northern Mediterranean in non-summer months, for example in the Gulf of Lions in April, which is characterised by colder waters than other areas surveyed in that month.

A Mediterranean-wide survey is scheduled to take place in the summer 2018 thanks to the long-standing initiative of ACCOBAMS (Agreement on the Conservation of Cetaceans in the Black Sea, Mediterranean Sea and Contiguous Atlantic Area). This regional survey will be extremely helpful to fill existing data gaps by collecting data synoptically with a consistent methodology. Unfortunately, this survey will not be sufficient to fill data gaps in non-summer months in which environmental conditions (and by extension cetacean distributions) are widely different. Achieving a representative sample of environmental space is critical for surveys intended for habitat-based density modelling of cetaceans. An analysis of environmental space early in the survey planning process—for example assessing the environmental representativeness of a set of potential survey designs—can help ensure model-based predictions of cetacean densities are derived with limited extrapolation for the study region.

The natural next step of this study is to incorporate cetacean observations obtained from the aggregated surveys to build models and predict cetacean densities across the Mediterranean Sea. The resulting density maps could be related to maps of human activities such as ship traffic and underwater noise, which are particularly harmful to cetaceans in the Mediterranean Sea^30,47^, in order to identify unsurveyed areas where populations may be more at risk. Predicted density maps could also be used for delineating areas of potentially high cetacean densities in poorly surveyed regions of the Mediterranean Sea, and thus be utilised as part of the Important Marine Mammal Area (IMMA) process of International Union for Conservation of Nature.

## Methods

### Study area

The Mediterranean Sea is a semi-enclosed water body connected to the Atlantic Ocean by the Strait of Gibraltar, to the Black Sea by the Bosphorus, and since 1869 to the Red Sea by the Suez Canal. It is divided into a western and an eastern basin by a central ridge between Sicily and the Tunisian-Libyan coast. The Mediterranean Sea is mainly characterised by narrow continental shelves, steep slopes and extensive abyssal plains. It includes a variety of submarine canyons, mostly located along the continental slopes in the north. It also includes approximately one hundred seamounts (Supplementary Fig. S15), known to affect the distribution of pelagic species, including cetaceans^48–50^. The Mediterranean is an oligotrophic sea characterised by salty and nutrient-poor waters^51^.

Circulation in the Mediterranean Sea is mainly driven by water flow through the Strait of Gibraltar, freshwater inputs from the main rivers (Nile, and to a lesser extent, Po, Rhone and Ebro), wind stress, and thermohaline and topographic features^52^. Atlantic Surface Water flows into the Mediterranean Sea through the Strait of Gibraltar and circulates in a cyclonic (counterclockwise) direction (Fig. 1). Water flow along the southern coasts generates short-lived mesoscale anticyclonic eddies (e.g., the eddy field off Algeria). To the north, water flow creates persistent cyclonic gyres (e.g., the Lions gyre) associated with upwelling of nutrient-rich waters that result in enhanced primary productivity^52^. As it moves eastward, surface water evaporates and becomes saltier, warmer and poorer in nutrients, resulting in a gradual decline in phytoplankton biomass and productivity from west to east^16,53^. As it becomes saltier and denser, the Atlantic Surface Water sinks in the Levantine Sea, returning westward as Levantine Intermediate Water before exiting into the Atlantic through the Strait of Gibraltar. During winter, water sinks in the Aegean, Adriatic and Ligurian seas and goes to the very bottom, creating the Mediterranean Deep Water^52^.

Phytoplankton biomass and primary production have marked seasonal cycles in the Mediterranean Sea^54,55^. Phytoplankton blooms are primarily initiated in winter and spring by wind stress, causing mixing and nutrient uplift to surface layers. Uplift of nutrients also occurs at cyclonic eddies. While blooms are markedly seasonal and intense in the northwestern basin (e.g., in the Gulf of Lions), they are often sporadic and subject to significant inter-annual variability in the eastern basin. Stratification occurs in summer, resulting in a lower and more homogeneous phytoplankton biomass across the Mediterranean Sea.

The spatial extent for this study corresponded to the entire Mediterranean Sea, excluding enclosed lagoons and estuaries (surface area: 2.5 million km^2^). The study area was projected to a custom Lambert Azimuthal Equal Area projection in ArcGIS 10.2.2 and gridded into 10 × 10 km cells for analysis, representing a compromise between the various resolutions of environmental covariates (see Supplementary Tables S1 and S2). The Mediterranean study area was divided into 8 distinct ecological subregions following previous studies^18,56^ (Fig. 2).

### Line transect surveys

We aggregated data from visual shipboard and aerial line transect surveys that followed distance sampling methodology, i.e., allowed the calculation of perpendicular distances to observed groups of cetaceans and thus the estimation of absolute abundances^57^. We considered both occasional large-scale surveys and recurrent small-scale surveys aimed at abundance estimation (homogeneous coverage probability was not a requirement for this analysis). We included only line transect surveys conducted after October 1997 to ensure the full set of environmental covariates was available (primary productivity data was available from October 1997; see Supplementary Table S2), and until April 2016.

### Spatiotemporal coverage of surveys

For each survey, we created in ArcGIS a shapefile of on-effort tracklines with associated latitude, longitude and date. We merged shapefiles of all survey tracklines and added them to a file geodatabase. We then intersected and spatially joined the tracklines to the 10 × 10 km grid of the study area. To examine the spatiotemporal coverage of surveys, we summed survey effort per grid cell in the entire study area and created subsets of surveys per month, year, and platform (ship or aircraft).

### Environmental covariates

We examined the environmental coverage of surveys with respect to four static covariates (depth, slope, distance to canyons, and distance to seamounts) and three dynamic covariates (sea surface temperature, primary productivity, and eddy kinetic energy) known to be important for cetaceans and likely to be included in future habitat-based density models (Supplementary Tables S1 and S2).

We created rasters of static and dynamic covariates (data sources detailed in Supplementary Tables S1 and S2) and resampled them to the 10 × 10 km grid of the study area (using nearest neighbour interpolation implemented in the Marine Geospatial Ecology Tools software^58^) in ArcGIS.

We used monthly contemporaneous and climatological resolutions for dynamic covariates^59^. For surveyed grid cells (i.e., representing the “available” dataset; see below), we used the month and year when the survey took place (contemporaneous resolution). For all grid cells of the study area (i.e., representing the “prediction” dataset; see below), we used a monthly climatological average describing long-term oceanographic conditions in the Mediterranean Sea. Doing so allowed us to compare surveyed oceanographic conditions with long-term oceanographic averages (rather than oceanographic conditions of a particular year). To obtain monthly climatological rasters, we averaged monthly rasters per grid cell from 1997–2016. These climatological means smooth out the inter-annual variability of oceanographic processes while maintaining the strong intra-annual variability which characterizes the Mediterranean Sea^52,54,55^.

### Environmental coverage of surveys

To analyse the environmental coverage of surveys, we examined two multivariate environmental metrics: the extent of extrapolation *versus* interpolation and the proportion of prediction points near available data points.

To evaluate the extent of extrapolation *versus* interpolation if models calibrated on the available survey data were used to predict cetacean densities in the entire Mediterranean Sea, we used the convex hull approach^37,60,61^. The convex hull of a set of points is defined as the smallest convex set that contains these points. Predictions inside the convex hull are interpolations while predictions outside the convex hull are extrapolations. The convex hull of a single covariate is the interval between the minimum and maximum data points (i.e., a univariate environmental envelope). The convex hull of two covariates is a polygon with vertices at the extreme points of the data points (i.e., a bivariate environmental envelope). A convex hull can be defined for any number of covariates, although visualization becomes difficult for more than two covariates and computational power starts to limit calculations for more than ten covariates^61^. As stressed by Authier *et al*. (2017), the assessment of the convex hull does not require model fitting (only effort and environmental covariate data are required)^37^.

Here, environmental characteristics of the surveyed grid cells during the month and year of each survey represented the “available” dataset and monthly climatological environmental conditions of all grid cells of the study area represented the “prediction” dataset. We considered the seven individual covariates enumerated above and the three following combinations of covariates:

- all static covariates: depth, slope, distance to seamounts, and distance to canyons;
- all dynamic covariates: sea surface temperature, primary productivity, and eddy kinetic energy;
- all static and dynamic covariates: depth, slope, distance to seamounts, distance to canyons, sea surface temperature, primary productivity, and eddy kinetic energy.

An examination of Spearman’s rank correlation coefficients^62^ showed that these covariates were not highly correlated (Spearman’s rho <|0.6|) in both available and prediction datasets.

To further quantify extrapolation in environmental space, we calculated Gower’s distances (*G*^2^) for the three combinations of covariates listed above. As for the convex hull, the calculation of Gower’s distances does not require model fitting. The Gower’s non-parametric distance between points *i* and *j* belonging to the available and prediction datasets, respectively, is defined as the average absolute distance between the values of these two points in each dimension, divided by the range of the data^61–63^. With *K* environmental dimensions, the Gower’s distance formula is as follows:

$${G}_{ij}^{2}=\frac{1}{K}\sum _{k=1}^{K}\frac{|{x}_{ik}-{x}_{jk}|}{{r}_{k}}$$

where *r*_*k*_ is the difference between the largest and the smallest values of the available dataset for the *k*_th_ covariate. Because of range standardization, covariates have equal contribution to the Gower’s distance. We considered a prediction point to be in the neighbourhood of available data points if it was situated within a radius of one geometric mean Gower’s distance of all pairs of available data points^61^. We stress that this definition of neighbourhood relates to the multivariate environmental space defined by the considered environmental covariates, not geographic space. The larger the proportion of prediction points near available data points in this multivariate environmental space (“proportion of data nearby” for brevity), the more reliable the extrapolation.

We used the WhatIf package (version 1.5-6)^64^ in R (version R-3.1.1)^65^ to calculate convex hulls and Gower’s distances. We projected the extent of extrapolation *versus* interpolation and the proportion of data nearby to geographic space for mapping.

### Data availability statement

The aggregated survey dataset and R code to reproduce the gap analysis are made available via the Dryad repository (10.5061/dryad.4pd33).

## Electronic supplementary material

Supplementary information
