# Supplementary material for: Assessing cetacean surveys throughout the Mediterranean Sea: a gap analysis in environmental space
Source: Sci Rep. 2018 Feb 15;8:3126. doi: 10.1038/s41598-018-19842-9 (PMC5814436; doi:10.1038/s41598-018-19842-9)
Supplement: Supplementary file 1 — Supplementary information [file 41598_2018_19842_MOESM1_ESM.doc]

Assessing cetacean surveys throughout the Mediterranean Sea: a gap analysis in environmental space

Laura Mannocci, Jason J. Roberts, Patrick N. Halpin, Matthieu Authier, Oliver Boisseau, Mohamed Nejmeddine Bradai, Ana Cañadas, Carla Chicote, Léa David, Nathalie Di-Méglio, Caterina M. Fortuna, Alexandros Frantzis, Manel Gazo, Tilen Genov, Philip S. Hammond, Drasko Holcer, Kristin Kaschner, Dani Kerem, Giancarlo Lauriano, Tim Lewis, Giuseppe Notarbartolo di Sciara, Simone Panigada, Juan Antonio Raga, Aviad Scheinin, Vincent Ridoux, Adriana Vella, Joseph Vella

SUPPLEMENTARY INFORMATION

SUPPLEMENTARY TABLES

Supplementary Table S1: Static covariates considered in this gap analysis and likely to be included in future habitat-based density models of cetaceans.

| Covariate (unit) | Original spatial resolution | Data source and processing details | Brief ecological rationale |
| --- | --- | --- | --- |
| Depth (m) | 30 arc seconds | SRTM30-PLUS global bathymetry (ftp://topex.ucsd.edu/pub/srtm30_plus/) | Cetaceans occur over the continental shelf (~0-200m), the continental slope (~200-2000m), and/or the abyssal plain (~>2000m). |
| Slope (°) | 30 arc seconds | Derived from SRTM30-PLUS global bathymetry using the Slope tool of Spatial Analyst in ArcGIS 10.2.2. | Cetaceans associate with steep slopes that are characterized by enhanced primary and secondary production and/or prey aggregation. |
| Distance to the nearest seamount (km) | 30 arc seconds | Derived from the global seamounts database ^1^. Both seamounts and knolls (smaller seamounts) were considered. A least cost path was used for the distance calculation to account for the complex coastline. | Cetaceans, especially deep divers, associate with seamounts. Seamounts affect water circulation (e.g., by trapping eddies) and generate upwelling, leading to increased primary and secondary production and/or prey aggregation. |
| Distance to the nearest submarine canyon (km) | 30 arc seconds | Derived from the global geomorphology database ^2^. A least cost path was used for the distance calculation to account for the complex coastline. | Cetaceans, especially deep divers, associate with submarine canyons. Currents, eddies and upwelling generated on the outer part of submarine canyons enhance biological production. |

Supplementary Table S2: Dynamic covariates considered in this gap analysis and likely to be included in future habitat-based density models of cetaceans.

| Covariate (abbreviation) (unit) | Original spatial resolution | Original temporal resolution | Temporal availability | Data source and processing details | Brief ecological rationale |
| --- | --- | --- | --- | --- | --- |
| Sea surface temperature (SST) (°C) | 0.2⁰ | Daily | 9/1/1991 - present | Group for High Resolution Sea Surface Temperature Level 4 CMC 2.0 SST from NASA (<http://podaac.jpl.nasa.gov/dataset/CMC0.2deg-CMC-L4-GLOB-v2.0>) ^3^. Monthly mean rasters were produced before resampling them to the grid of the study area. | Cetaceans often associate with water masses characterized by distinct surface temperatures. Surface temperature influences circulation in the upper water column and the distribution of ectothermic organisms that constitute the cetaceans’ prey. |
| Primary productivity (PP) (mg C m^-2^ day^-1^) | 9 km | 8 days | 9/14/1997 - present | Vertically generalized production model from Oregon State University (http://www.science.oregonstate.edu/ocean.productivity/) ^4^. A median filter was applied. Monthly mean rasters were produced before resampling them to the grid of the study area. | Cetaceans often occur in areas of enhanced primary production. Enhanced primary production might cause enhanced secondary production, thus increasing the availability of prey for cetaceans. |
| Eddy kinetic energy (EKE) cm² s-² | 0.125⁰ | monthly | 1/1/1993 - present | Derived from AVISO DT-MSLA geostrophic currents from the Mediterranean regional model (http://www.aviso.altimetry.fr/en/data/products/sea-surface-height-products/regional/madt-mediterranean-sea.html). | Cetaceans frequently associate with mesoscale eddies that enhance primary production owing to the upwelling of nutrient-rich waters (cyclonic mesoscale eddies), and aggregate prey. |

SUPPLEMENTARY FIGURES


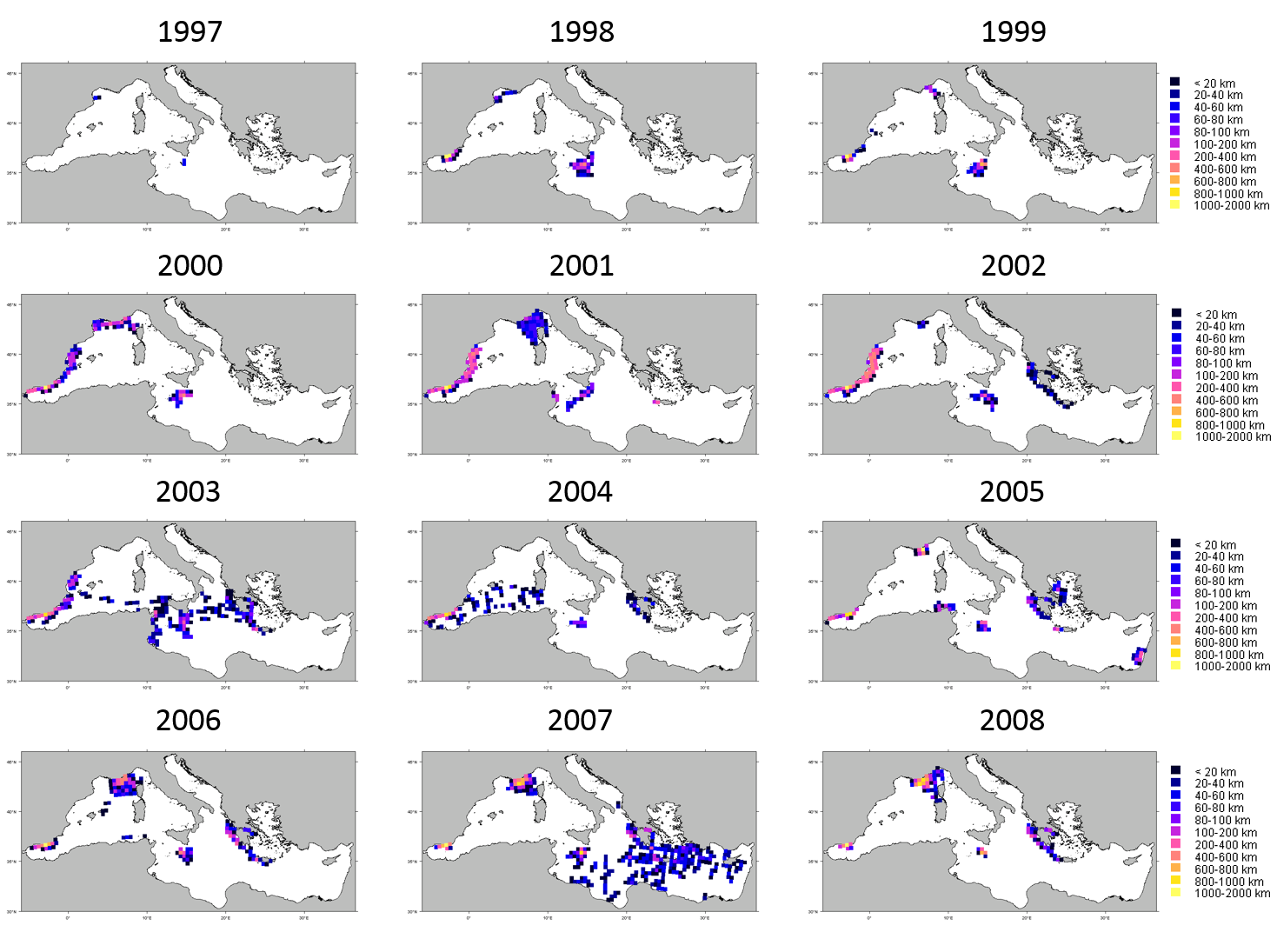

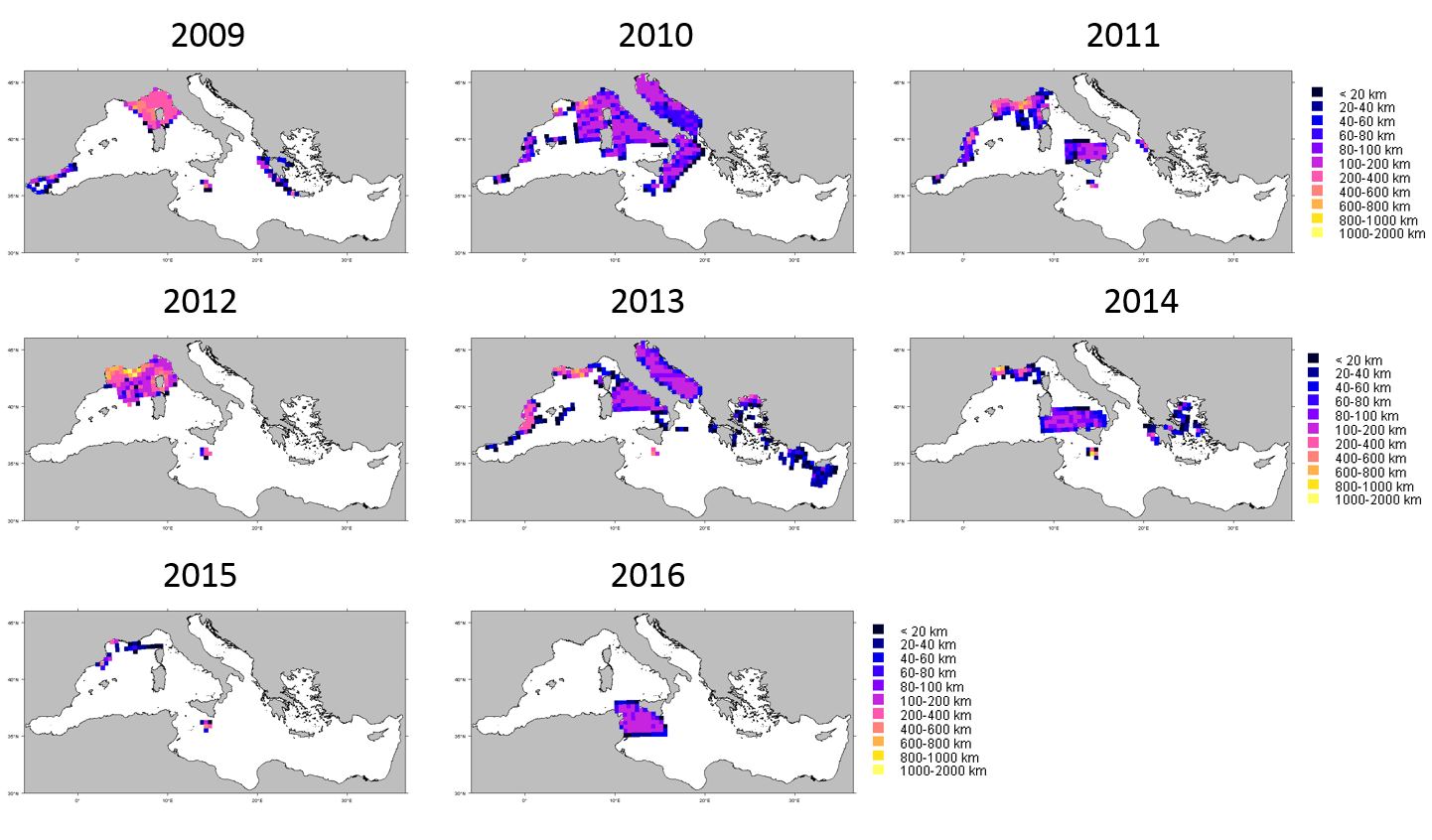


Supplementary Figure S1: Geographic coverage of survey effort per year in the Mediterranean Sea. Effort was aggregated on a 20 × 20 km grid for visualization. The color scale represents effort in km per 20 × 20 km grid cell and is the same for all maps. Blank cells represent zero effort. The maps were generated with R (<https://www.r-project.org/>) (version 3.1.1).


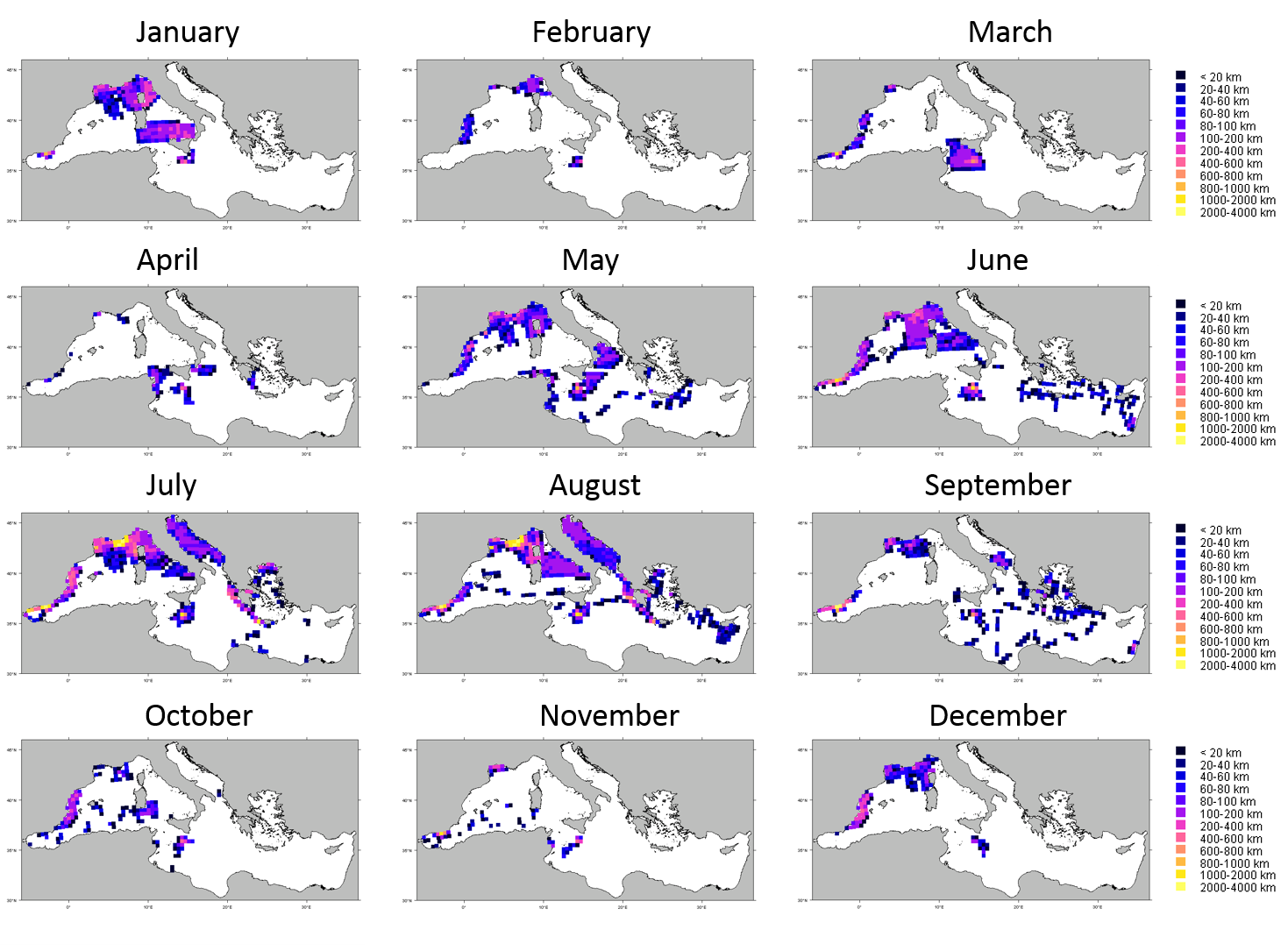


Supplementary Figure S2: Geographic coverage of survey effort per month in the Mediterranean Sea. Effort was aggregated on a 20 × 20 km grid for visualization. The color scale represents effort in km per 20 × 20 km grid cell and is the same for all maps. Blank cells represent zero effort. The maps were generated with R (<https://www.r-project.org/>) (version 3.1.1).

Supplementary Figure S3: Kernel density distribution of considered static covariates in the Mediterranean Sea. A rug plot of covariate values in the Mediterranean is superimposed on the x-axis. Covariate values spanned by surveys (i.e., corresponding to situations of interpolation) are indicated in yellow. Covariate values lower/higher than values spanned by surveys (i.e., corresponding to situations of extrapolation) are indicated in blue/red.


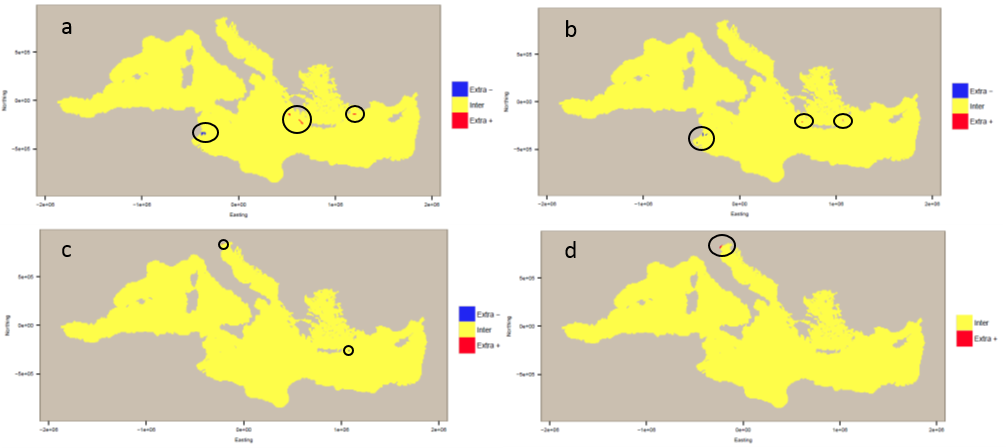


Supplementary Figure S4: Extent of extrapolation versus interpolation if models calibrated on the available survey data were used for prediction across the Mediterranean Sea. (a): model including depth only; (b): model including slope only; (c): model including distance to seamounts only; (d): model including distance to canyons only. Cells where extrapolation to lower/higher values occurred are indicated in blue/red. Cells where interpolation occurred are indicated in yellow. Extrapolation cells are encircled to enhance their visibility. The maps were generated with R (<https://www.r-project.org/>) (version 3.1.1).


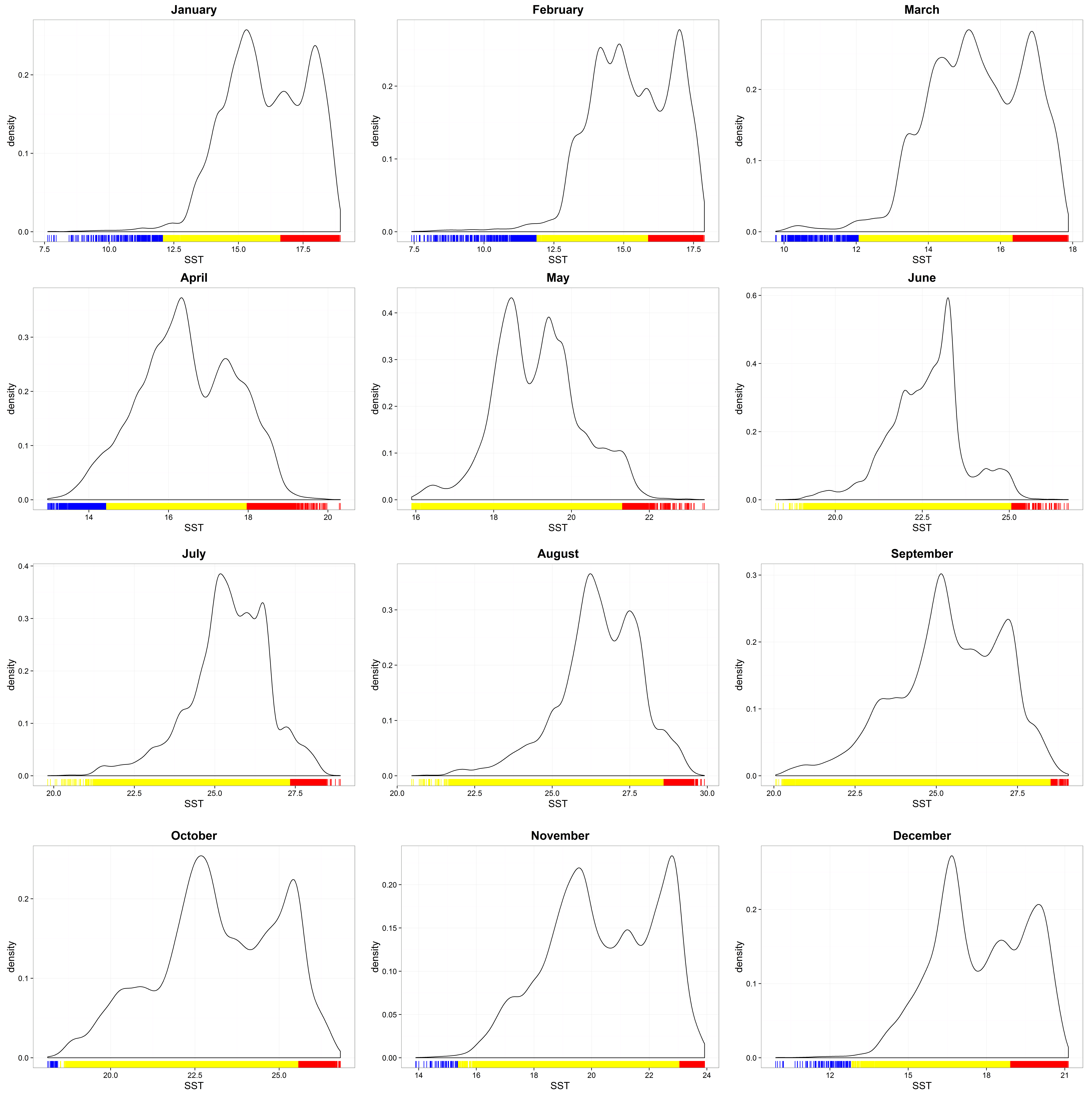


Supplementary Figure S5: Kernel density distributions of sea surface temperature in the Mediterranean Sea per month. Rug plots of covariate values in the Mediterranean Sea are superimposed on the x-axes. Covariate values spanned by surveys (i.e., corresponding to situations of interpolation) are indicated in yellow. Covariate values lower/higher than values spanned by surveys (i.e., corresponding to situations of extrapolation) are indicated in blue/red. Covariate values at surveyed locations were sampled at a monthly contemporaneous resolution. Covariate values in the Mediterranean Sea were sampled at a monthly climatological resolution (see Methods).

Supplementary Figure S6: Extent of extrapolation versus interpolation if a model including sea surface temperature calibrated on the available survey data was used for prediction across the Mediterranean Sea. Cells where extrapolation to lower/higher values occurred are indicated in blue/red. Cells where interpolation occurred are indicated in yellow. The maps were generated with R (<https://www.r-project.org/>) (version 3.1.1).


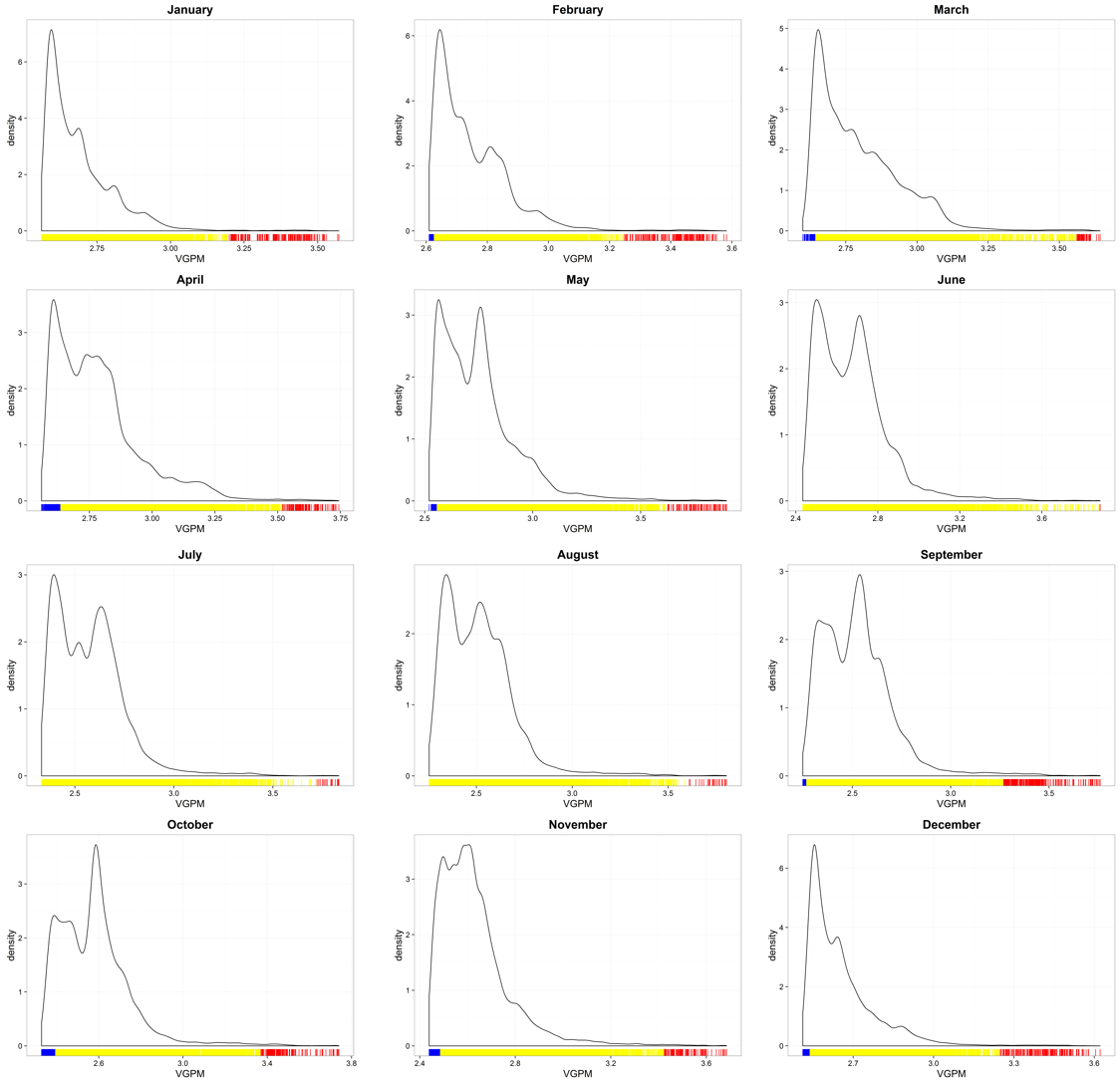


Supplementary Figure S7: Kernel density distributions of primary productivity in the Mediterranean Sea per month. Rug plots of covariate values in the Mediterranean are superimposed on the x-axes. Covariate values spanned by surveys (i.e., corresponding to situations of interpolation) are indicated in yellow. Covariate values lower/higher than values spanned by surveys (i.e., corresponding to situations of extrapolation) are indicated in blue/red. Covariate values at surveyed locations were sampled at a monthly contemporaneous resolution. Covariate values in the Mediterranean Sea were sampled at a monthly climatological resolution (see Methods).

Supplementary Figure S8: Extent of extrapolation versus interpolation if a model including primary productivity calibrated on the available survey data was used for prediction across the Mediterranean Sea. Cells where extrapolation to lower/higher values occurred are indicated in blue/red. Cells where interpolation occurred are indicated in yellow. The maps were generated with R (<https://www.r-project.org/>) (version 3.1.1).


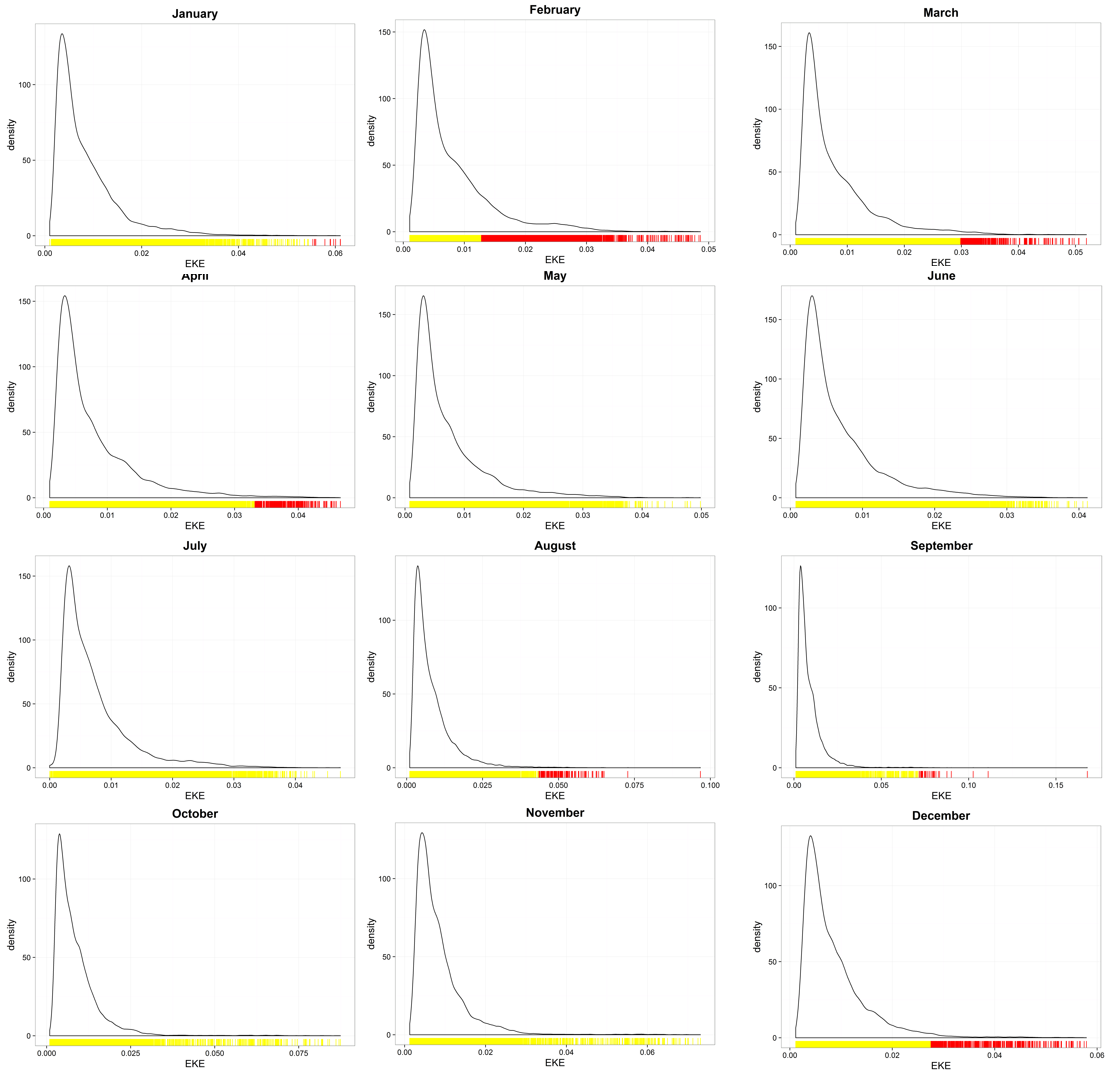


Supplementary Figure S9: Kernel density distributions of eddy kinetic energy in the Mediterranean Sea per month. Rug plots of covariate values in the Mediterranean Sea are superimposed on the x-axes. Covariate values spanned by surveys (i.e., corresponding to situations of interpolation) are indicated in yellow. Covariate values lower/higher than values spanned by surveys (i.e., corresponding to situations of extrapolation) are indicated in blue/red. Covariate values at surveyed locations were sampled at a monthly contemporaneous resolution. Covariate values in the Mediterranean Sea were sampled at a monthly climatological resolution (see Methods).

Supplementary Figure S10: Extent of extrapolation versus interpolation if a model including eddy kinetic energy calibrated on the available survey data was used for prediction across the Mediterranean Sea. Cells where extrapolation to lower/higher values occurred are indicated in blue/red. Cells where interpolation occurred are indicated in yellow. The maps were generated with R (<https://www.r-project.org/>) (version 3.1.1).

Supplementary Figure S11: Extent of extrapolation (dark blue) versus interpolation (yellow) if a model including all considered dynamic covariates calibrated on the available survey data was used for prediction across the Mediterranean Sea. Cells where extrapolation occurred are indicated in dark blue. Cells where interpolation occurred are indicated in yellow. The maps were generated with R (<https://www.r-project.org/>) (version 3.1.1).

Supplementary Figure S12: Proportion of prediction points near available data points in the multivariate environmental space defined by all considered dynamic covariates if a model including all dynamic covariates calibrated on the available survey data was used for prediction across the Mediterranean Sea. Dark blue/yellow represents areas where predictions would potentially be unreliable/reliable. The definition of neighborhood in multivariate environmental space is provided in the Methods. The maps were generated with R (<https://www.r-project.org/>) (version 3.1.1).

Supplementary Figure S13: Extent of extrapolation (dark blue) versus interpolation (yellow) if a model including all considered static and dynamic covariates calibrated on the available survey data was used for prediction across the Mediterranean Sea. Cells where extrapolation occurred are indicated in dark blue. Cells where interpolation occurred are indicated in yellow. The maps were generated with R (<https://www.r-project.org/>) (version 3.1.1).

Supplementary Figure S14: Proportion of prediction points near available data points in the multivariate environmental space defined by all considered static and dynamic covariates if a model including all static and dynamic covariates calibrated on the available survey data was used for prediction across the Mediterranean Sea. Dark blue/yellow represents areas where predictions would potentially be unreliable/reliable. The definition of neighborhood in multivariate environmental space is provided in the Methods. The maps were generated with R (<https://www.r-project.org/>) (version 3.1.1).


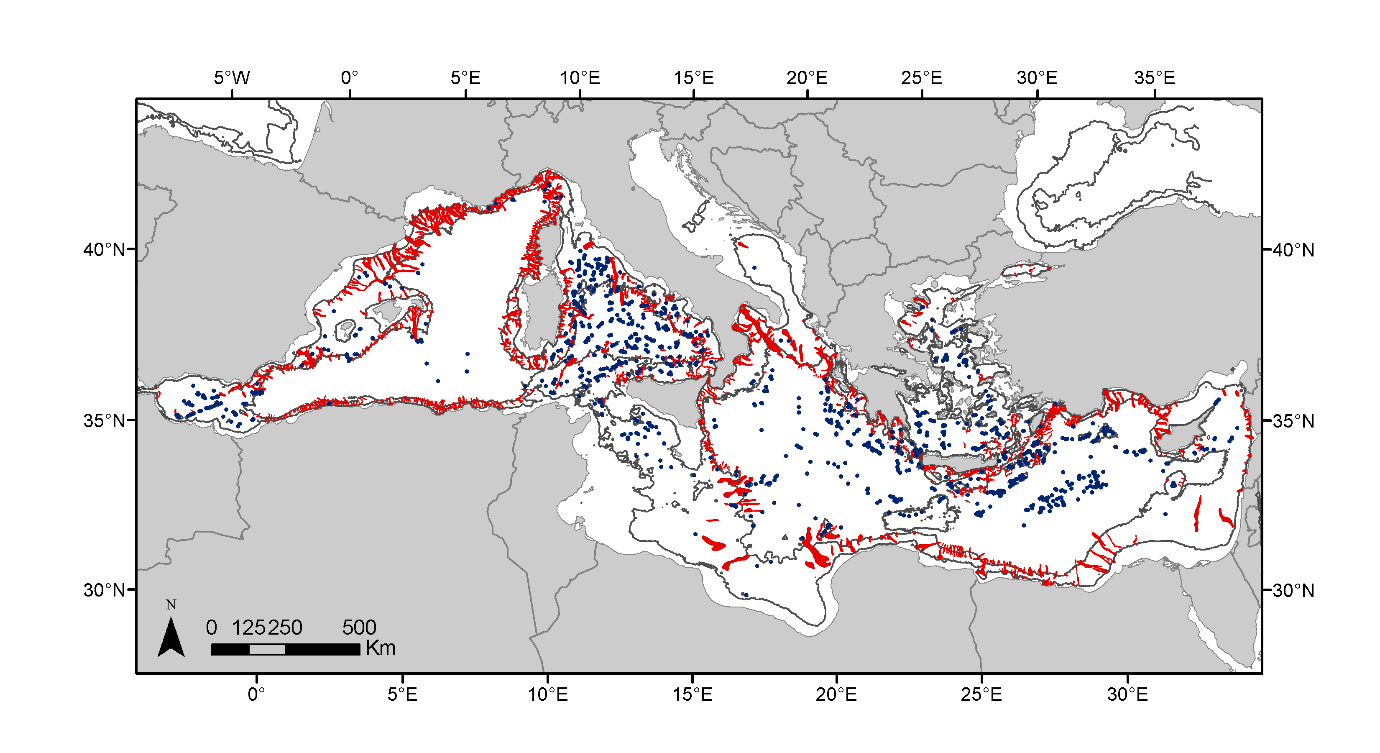


Supplementary Figure S15: Locations of submarine canyons (red) and seamounts (blue) throughout the Mediterranean Sea obtained from the global databases of Yesson et al. (2011) ^1^ and Harris et al. (2014) ^2^, respectively. The 200 m and 2000 m isobaths are indicated. The map was generated with ArcGIS (http://desktop.arcgis.com/en/) (version 10.2.2).

REFERENCES

1. Yesson, C., Clark, M. R., Taylor, M. L. & Rogers, A. D. The global distribution of seamounts based on 30 arc seconds bathymetry data. *Deep Sea Res. Part Oceanogr. Res. Pap.* **58,** 442–453 (2011).

2. Harris, P. T., Macmillan-Lawler, M., Rupp, J. & Baker, E. K. Geomorphology of the oceans. *Mar. Geol.* **352,** 4–24 (2014).

3. Brasnett, B. The impact of satellite retrievals in a global sea-surface-temperature analysis. *Q. J. R. Meteorol. Soc.* **134,** 1745–1760 (2008).

4. Behrenfeld, M. J. & Falkowski, P. G. Photosynthetic rates derived from satellite-based chlorophyll concentration. *Limnol. Oceanogr.* **42,** 1–20 (1997).
